# Accuracy and prognostic ability of the SARC-F questionnaire and Ishii's score in the screening of sarcopenia in geriatric inpatients

**DOI:** 10.1590/1414-431X20198204

**Published:** 2019-08-29

**Authors:** Min Li, Yan Kong, Hongcun Chen, Aiqin Chu, Guiqi Song, Yan Cui

**Affiliations:** 1 School of Humanities and Social Science, University of Science and Technology of China, Hefei, Anhui, China; 2 The First Affiliated Hospital of USTC, Division of Life Sciences and Medicine, University of Science and Technology of China, Hefei, Anhui, China; 3 Department of General Surgery, The Second People's Hospital of Hefei, Hefei Hospital Affiliated to Medical University of Anhui, Hefei, Anhui, China; 4 School of Nursing, Nanjing Medical University, Nanjing, China

**Keywords:** Sarcopenia, Muscle mass, Geriatric inpatients, Screening, Score

## Abstract

Sarcopenia remains poorly managed in clinical practice due to the lack of simple and accurate screening tools. This study aimed to identify the cutoff values of the SARC-F questionnaire and Ishii's score using the variables age, grip strength, and calf circumference in older inpatients in China to compare the accuracy of the two methods and to explore their predictive ability for adverse outcomes (rehospitalization, falls, fracture, and death). Hospitalized patients (n=138) aged ≥60 years were included. The accuracy of the two tools was evaluated using the reference diagnosis recommended by the Asian Working Group on Sarcopenia (assessing patients with measurements of muscle mass, handgrip strength, and usual gait speed). Follow-up data were obtained by telephone and clinical visits combined with the inpatient medical record system after discharge for at least one year. The results showed that the SARC-F score reached the highest Youden's index when a score of 3 was set as the cutoff value. Ishii's score presented a higher accuracy than SARC-F (area under the receiver operating curve: 0.78 *vs* 0.64, P=0.01). The Kaplan-Meier survival analysis demonstrated a higher cumulative incidence of rehospitalization in sarcopenic individuals compared to non-sarcopenic individuals according to SARC-F (log-rank test, P<0.001). Cox analysis revealed that SARC-F was an independent risk factor for rehospitalization (adjusted hazard ratio: 4.23, 95%CI: 2.12-9.79, P<0.001). The SARC-F and Ishii's scores might facilitate the early detection of sarcopenia and help identify older adults at risk for adverse outcomes in clinical practice.

## Introduction

Sarcopenia is characterized by progressive and generalized loss of skeletal muscle mass and strength with an increased risk of adverse outcomes such as physical disability, falls, fractures, poor quality of life, and death (1–3). The pathogenesis of sarcopenia is not clear, although some theories have proposed that anorexia associated with aging, lack of physical exercise, changes in endocrine function, cytokine imbalance, apoptosis, genetic influences, and mitochondrial dysfunction contribute to the development of sarcopenia (4–6). The decrease in muscle mass can be masked by an increase in weight and fat mass (7), especially in sarcopenic obesity, a condition of a high fat mass/low fat-free mass combination (8). Therefore, the early identification of the syndrome is essential to detect high-risk older adults who could benefit from intensive and multidisciplinary management to reduce the risk of adverse outcomes. The assessment techniques for sarcopenia include measurements of muscle mass, muscle strength, and muscle function. High costs, inconvenience, and/or radiological characteristics have restricted the use of magnetic resonance imaging, computed tomography, and dual-energy X-ray absorptiometry (DXA) for the assessment of muscle mass in the hospital setting, therefore precluding the general screening of sarcopenia (9,10). Simple, secure, and inexpensive screening tools with good performance would be convenient and helpful for the medical staff.

Currently, different screening tools are available. SARC-F is a rapid questionnaire to screen for sarcopenia using self-reported information about falls, mobility, and strength (11). Ishii's score is also a rapid screening test for sarcopenia. This test requires simple measures, including grip strength and calf circumference (12). The two methods might facilitate the extensive and rapid screening of geriatric patients in China due to their ease of use. Although the two methods have cutoff values defined in previous studies, the best thresholds for Chinese inpatients remain uncertain. In addition, a few studies have compared the accuracy of the two methods (13,14): Ishii's formula showed the best sensitivity and the largest area under the receiver operating curve (AUROC) to identify sarcopenia in older adults when compared with five different sarcopenia diagnostic definitions widely used, including SARC-F (14). Although previous studies have shown that these quick and simple screening methods predicted the occurrence of adverse outcomes such as rehospitalizations, falls, fractures, and death (9,15–21), their prognostic power remains unclear. Therefore, this study aimed to evaluate and compare the accuracy of the SARC-F and Ishii's scores in the screening for sarcopenia and to investigate their predictive value for adverse outcomes in hospitalized Chinese older adults.

## Material and Methods

### Participants

This study was conducted at the Body Composition Laboratory of the Geriatric Medical Healthcare Center at The First Affiliated Hospital of the University of Science and Technology of China (Anhui Provincial Hospital) in Hefei, China. Data were collected from a convenience sample of patients admitted to the geriatric acute care ward because of acute exacerbation of various chronic non-communicable diseases (cardiovascular diseases, neurological diseases, diabetes mellitus, osteoarthritis, depression, cancer, etc.). Assessment of bone mineral density by dual-energy X-ray absorptiometry (DXA) was a part of their medical examination in the Body Composition Laboratory where the study was performed.

A total of 138 inpatients were recruited between January 2015 and March 2016. The inclusion criterion stated that the patients were ≥60 years with the ability to perform all the body composition tests. The exclusion criteria were as follows: patients who were suffering from terminal illness or had metal medical devices in the body, patients with bone deformity (severe hump, scoliosis), depressed edema, rheumatoid arthritis, hand osteoarthritis, carpal tunnel syndrome, and patients who were administered medications, such as diuretics, that would affect body composition tests. This study was carried out in accordance with the Helsinki Declaration and was approved by the Ethics Committee of the hospital. All participants were fully informed, and signed informed consent was obtained.

### Methods

Patient information including gender, age, height, weight, physical activity level, and tobacco and alcohol use was collected. Clinical data were extracted from electronic medical records. Medical history was based on patients’ reports as well as the medical records examined retrospectively. The subjects were wearing only underwear after fasting 2–3 h and were asked to empty the bladder and remove all metal accessories and jewelry before measurements, which were taken in the morning and before administration of intravenous infusions.

### Screening for sarcopenia using the SARC-F questionnaire and Ishii's score

The SARC-F questionnaire was developed as a rapid diagnostic tool for sarcopenia. Five components closely related to functional status were self-reported by the older individuals: strength, assistance with walking, rising from a chair, climbing stairs, and falls, which were scored between 0 and 2, with higher scores being suggestive of sarcopenia. The score ranged from 0–10 (11).

Three variables were used to calculate Ishii's score: age, grip strength (kg), and calf circumference (cm). The grip strength measurement was done with a digital grip strength dynamometer (model: EH101, Xiangshan Instruments, China). The measurements were conducted two times using the dominant hand, and the higher of the two trials was used in the analysis. Calf circumference was measured at the maximum circumference of the non-dominant leg with the subjects' legs bent 90° at the knee. The formulas to calculate the score were as follows (12): Score in men = 0.62 (age – 64) – 3.09 (grip strength – 50) – 4.64 (calf circumference – 42), Score in women = 0.80 (age – 64) – 5.09 (grip strength – 34) – 3.28 (calf circumference – 42). In the study by Ishii et al. (12), the value of 105 for men and 120 for women provided the maximal sensitivity and specificity.

### Diagnostic criteria for sarcopenia

Sarcopenia was diagnosed with reduced muscle mass accompanied by decreased grip strength and/or gait speed. The reference standard used for evaluating the accuracy of the two methods was recommended by Asian Working Group on Sarcopenia (AWGS), which included measuring the muscle mass, grip strength, and gait speed (2).

### Muscle mass measurement

A fan-beam DXA densitometer (GE Lunar Prodigy, GE Healthcare, USA; enCORE™ 2008 software ver. 12.x) was used to measure body composition, including bone mineral content, fat mass, and lean soft tissue mass. The bone mineral-free lean soft tissue mass was used as an estimate of the muscle mass (kg) (22,23). The height-adjusted skeletal muscle mass (appendicular skeletal muscle mass/height^2^) was used to evaluate the muscle mass, and the suggested DXA cutoff values were 7.0 kg/m^2^ in men and 5.4 kg/m^2^ in women (2). Quality control procedures were performed daily before scanning.

### Measurement of muscle strength

AWGS suggests using handgrip strength as a feasible and convenient measure of muscle strength and recommends <26 kg for men and <18 kg for women as low handgrip strength (2).

### Measurement of physical performance

AWGS recommends a 6-m usual gait speed for measuring physical performance, with the cutoff of <0.8 m/s^2^ (2). Usual gait speed was measured by the subjects walking 6 m at usual speed. There were two meters of acceleration distances before the start point and two meters of deceleration distances after the end point.

### Follow-up for adverse outcomes

Follow-up data were obtained by telephone and clinical visits combined with the inpatient medical record system. The data were collected monthly by independent investigators who had not participated in inpatient enrollment for this study. Loss to follow-up was considered when participants did not have a new entry in the medical record after discharge and if the participants could not be reached by at least 10 telephone calls at different times during the follow-up period. The follow-up time was calculated from discharge to ensure that all patients were followed up for at least one year. The deadline for the follow-up was March 31, 2017. Patients who had not been hospitalized again before that date and who had been lost to follow-up were treated as censored data. The primary endpoint was rehospitalization defined as an event of hospital admission due to the same diagnosis and with a minimum 14-day interval from the discharge. The secondary endpoints included falls, fractures, and death.

### Statistical analysis

SPSS software (version 19.0, USA) and MedCalc (version 18.11.3, free trial, Belgium) were used for statistical analysis. Continuous variables are reported as means±SD. The Shapiro-Wilk test was used to assess normality. The differences in the homogeneity of variance among the groups were compared using the Mann-Whitney U test or independent sample *t*-tests. Dichotomous variables are reported as the number (percentage), and the χ^2^ test was used to compare the differences between the groups. The accuracy of the scores was evaluated using the sensitivity, specificity, Youden’s index, and AUROC. The optimal cutoff values for the two screening tools in the study population were analyzed by Youden’s index. The SARC-F and Ishii's scores were dichotomized according to these optimal cutoff values for detecting sarcopenia in the sample, and then the difference between the AUROC of the two methods was calculated using the nonparametric method developed by DeLong et al. (24). Kaplan-Meier analysis with the log-rank test was used to compare the difference between the sarcopenia and non-sarcopenia groups according to the SARC-F and Ishii's scores. Demographic factors (age and gender) and clinical variables, including body mass index (BMI), gait speed, physical activity level (25), smoking and drinking status, Charlson comorbidities index, grip strength, calf circumference, number of medications in use, and sarcopenia according to the Ishii's score and SARC-F were considered in the univariate Cox analysis. All variables with a P value of <0.05 in the univariate analysis were included in the multivariate Cox regression analyses. Two multivariate Cox regression models were built using the forced entry method to assess the association of sarcopenia, defined according to SARC-F or the Ishii's formula, with rehospitalization. Gender and age were excluded from the entry parameter, because they were used in the calculation of Ishii's score. P<0.05 was considered statistically significant.

## Results

### Characteristics of the study participants

A total of 138 older adults were included in the present study, 35 of them were diagnosed with sarcopenia according to the criteria recommended by AWGS, and 23 and 36 were classified as sarcopenic according to SARC-F and the Ishii's score, respectively. The characteristics are summarized in Table 1.

**Table 1.** Comparison of characteristics and clinical features of the participants.

Items	AWGS diagnosis (reference diagnosis)		P value
	Non-sarcopenia (n=103)	Sarcopenia (n=35)	
Age (years)	68.9±8.5	74.5±9.7	<0.001*
Men, [n (%)]	53 (76.8)	16 (23.2)	0.70**
BMI (kg/m^2^)	24.4±2.9	21.7±3.5	<0.001*
CCI [mean (quartile P_25_, P_75_)]	7.0 (5, 8)	6.9 (6, 8)	0.58***
Grip strength (kg)			
Men	32.7±7.4	23.4±3.7	<0.001*
Women	20.2±4.7	16.2±4.4	<0.001*
Gait speed (m/s)	1.0±0.3	0.7±0.3	<0.001*
Calf circumference (cm)	34.7±3.2	31.4±2.9	<0.001*

Data are reported as mean±SD, unless otherwise indicated. AWAGS: Asian Working Group on Sarcopenia; BMI: body mass index; CCI: Charlson comorbidity index. *Independent samples *t*-test; **Pearson χ^2^ test; ***Mann-Whitney U test.

### Accuracy of Ishii's score

**Figure 1.**
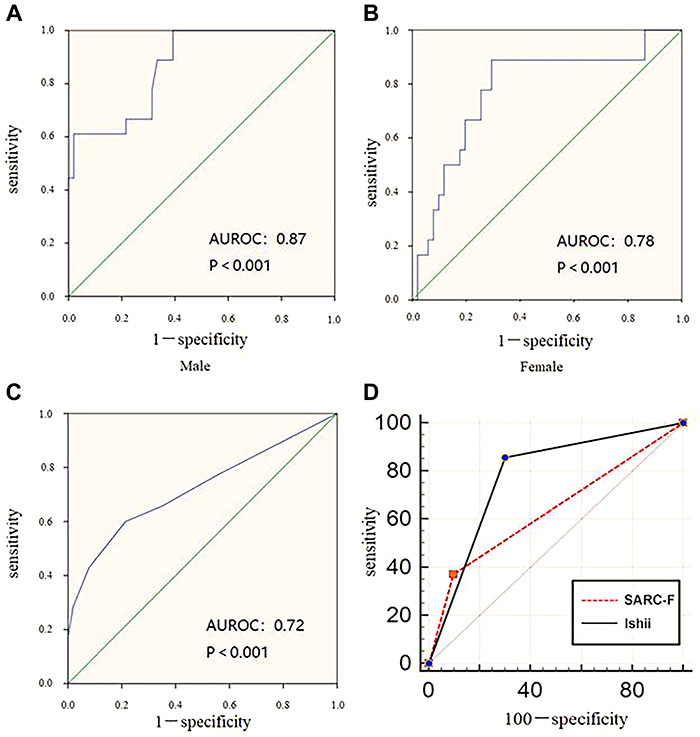
Receiver operating curve (ROC) of Ishii's score in males (**A**) and females (**B**). ROC for the SARC-F score (**C**). Comparison of ROC between the SARC-F and Ishii's scores for the screening for sarcopenia (**D**).

The AUROC was 0.87, 95% confidence interval (CI): 0.78-0.96, P<0.001 for males, Figure 1A, while it was 0.78, 95%CI: 0.65-0.91, P<0.001 for females, Figure 1B. Men reached the highest Youden’s index at 105, with corresponding sensitivity and specificity values of 88.9% and 70.6%, respectively. Women reached the highest Youden’s index at 120, and the corresponding sensitivity and specificity were 77.8 and 68.6%, respectively.

### Accuracy of the SARC-F questionnaire

The AUROC was 0.72 with 95%CI: 0.61-0.83, P<0.001 (Figure 1C) and the cutoff value of 3 reached the highest Youden's index. The corresponding sensitivity and specificity were 42.9 and 92.2%, respectively.

### Comparison of the accuracy of the SARC-F and Ishii's scores to identify sarcopenia

All patients were categorized by the SARC-F and Ishii's scores into sarcopenia and non-sarcopenia groups. The accuracy of the grouping was compared using the recommendation of AWGS as a reference. SARC-F presented a lower AUROC than Ishii's score (AUC 0.64 *vs* 0.78), and the difference was 0.14, 95%CI: 0.03-0.25, z=2.48, P=0.01 (Figure 1D).

### Prognostic ability of the SARC-F and Ishii's scores for adverse outcomes

Of the 138 patients, 12 (8.7%) were lost to follow-up. No significant differences were detected in the demographic characteristics and medical history between the 126 patients who completed the follow-up and the 12 patients who were lost to follow-up. The incidence of adverse outcomes in each group (sarcopenic *vs* non-sarcopenic) according to SARC-F was 26.1 (n=6) *vs* 2.6% (n=3) for falls, 17.4% (n=4) *vs* 0 (n=0) for fractures, 39.1 (n=9) *vs* 17.4% (n=20) for rehospitalizations, and 8.7% (n=2) *vs* 0 (n=0) for deaths. The incidence of adverse outcomes in each group according to Ishii's score was 9.8 (n=6) *vs* 3.9% (n=3) for falls, 4.9 (n=3) *vs* 1.3% (n=1) for fractures, 27.9 (n=17) *vs* 15.6% (n=12) for rehospitalizations, and 3.3% (n=2) *vs* 0 (n=0) for deaths. The low rate of these outcomes hindered possible comparisons between groups.

### Comparison of the interval times to rehospitalization

**Figure 2.**
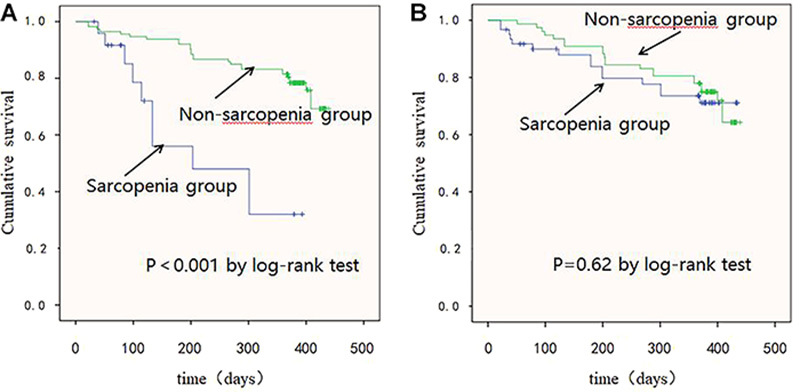
Kaplan-Meier analysis for rehospitalization. **A**: Dichotomy by the SARC-F score, log-rank test χ^2^=18.39, P<0.001. **B**: Dichotomy by Ishii's score, log-rank test χ^2^=0.24, P=0.62.

The difference between the survival curves between the sarcopenia and non-sarcopenia groups according to SARC-F was statistically significant. The sarcopenia group exhibited a shorter time interval than the non-sarcopenia group (log-rank test, P<0.001) (Figure 2A).

The difference between the survival curves between the sarcopenia and non-sarcopenia groups according to Ishii's score was not statistically significant (log-rank test, P=0.62) (Figure 2B).

### Cox proportional hazard analysis for rehospitalization

Rehospitalization was the observational index. A single-factor Cox regression analysis indicated that age, gait speed, physical function, SARC-F value, Ishii's score grouping, number of medications (prescription drugs after discharge), and Charlson comorbidities index (CCI) were significantly correlated with rehospitalization (Table 2). All seven significant factors in the single-factor Cox analysis were entered into the forward conditional regression analysis. From the components of the two Cox regression multivariate models, SARC-F score, CCI, and gait speed were independent factors influencing the rehospitalization of older adults (Table 3). In the case of the other constant factors, the risk of rehospitalization increased by 3.23-fold when the SARC-F score increased by 1 point, and the risk increased by 2.01-fold when the CCI increased by 1 point. Gait speed was a protective factor against rehospitalization risk.

**Table 2.** Results of univariate Cox proportional hazard analysis for rehospitalization.

Variables	HR	95% CI	P value
Age	1.06	1.01–1.12	0.002
Physical activity level	0.22	0.07–0.65	0.01
Sarcopenia according to Ishii's formula	1.55	1.26–1.90	<0.001
Number of medications	1.18	1.01–1.40	0.04
CCI	1.34	1.08–1.64	0.01
Gait speed	0.16	0.03–0.75	0.02
SARC-F score	5.44	2.26–13.11	<0.001

CCI: Charlson comorbidities index; HR: hazard risk. Number of medications included the prescription drugs after discharge; physical activity level was obtained from the International Physical Activity Questionnaire and was categorized into 3 levels (low, moderate, high) according to the proposed cutoff values 25.

**Table 3.** Results of multivariate Cox proportional hazard analysis for rehospitalization.

	Model 1		Model 2	
	HR (95% CI)	P value	HR (95% CI)	P value
Age	1.13 (0.88–1.13)	0.71	–	–
Gender	0.79 (0.56–1.43)	0.32	–	–
SARC-F score	4.23 (2.12–9.79)	0.001	–	–
Ishii’s score	–	–	1.34 (0.61–1.73)	0.28
CCI	3.01 (1.29–6.32)	0.02	2.69 (1.69–4.54)	0.03
Active	1.53 (0.90–2.14)	0.37	1.03 (0.84–1.63)	0.33
Gait speed	0.01 (0.001–0.39)	<0.001	0.01 (0.001–0.26)	<0.001

The forced entry method was used in the COX multivariate analysis. CCI: Charlson comorbidities index. Age and gender were excluded from Model 2, because they were used in the calculation of Ishii's score.

## Discussion

### Accuracy of SARC-F in screening for sarcopenia

Despite its clinical importance, sarcopenia remains poorly managed in routine clinical practice. Malmstrom and Morley suggested that SARC-F ≥4 could predict sarcopenia (11). In this study, compared to a value of 4, the cutoff value of 3 indicated higher sensitivity, lower specificity, and reached the optimal Youden’s index. The selection of a cutoff value should be based on specific application purposes, for example, whether high sensitivity or specificity is desired. Therefore, we suggested 3 as the cutoff value for hospitalized Chinese patients at high risk to raise the detection rate of sarcopenia. In a population survey, the cutoff value of 4 is preferable to accurately identify those at high risk and exclude those at low risk.

Woo et al. (26) reported that the sensitivity of SARC-F was <10% and that the specificity was >90% in a survey of 4,000 older adults ≥65 years of age. The sensitivity in the current study was higher than that described by Woo et al. (26). This difference in the sensitivity was attributed to differences in the study population: hospitalized individuals *vs* community individuals. Strikingly, the sarcopenia prevalence in the two groups might have affected the sensitivity. Despite these differences in sensitivity, SARC-F had high specificity, rendering a low rate of misdiagnosis. Therefore, SARC-F is a simple and quick method for screening sarcopenia in primary health institutions and can detect high-risk patients with sarcopenia for further examination and intervention, which might contribute to the prevention of adverse outcomes.

### Accuracy of Ishii's score in screening for sarcopenia

In this study, we achieved identical cutoff values for Ishii's score to a previous study on screening for sarcopenia (27). One hundred and five for men and 120 for women were the optimal cutoff values for Ishii's score, and the corresponding sensitivity and specificity were high. The present and previous studies were performed in the Asian population and using the AWGS as the reference standards, which might be the reason for identical cutoff values.

### Comparison of the accuracy of the SARC-F and Ishii’s scores

The SARC-F and Ishii's scores showed their strengths in the screening of sarcopenia in hospitalized older adults: high specificity of SARC-F prevents unnecessary and inconvenient investigations for those not at-risk, but in an overall comparison, the AUROC of Ishii's score was significantly higher than that of SARC-F. The potential reasons might be that, first, Ishii's score was developed in an Asian population as this study and second, Ishii's formula included two measurable indicators, grip strength and calf circumference, which were similar to the variables used in the reference diagnostic criteria: grip strength and muscle mass.

Previous studies have reported high detection rates (21.4-79.8%) of sarcopenia using different criteria and cutoff values in hospitalized older patients (28,29). The sarcopenia detection rate was high in this study, demanding the attention of the clinical staff. Furthermore, irrespective of the cutoff, the clinical staff should pursue multidisciplinary interventions for the patients identified as having a high risk of sarcopenia to reduce the risk of adverse outcomes and improve functional status after acute care.

### Ability to predict poor outcomes of sarcopenia

The prediction of the risk of adverse outcomes is a major factor in evaluating the performance of screening scores. In a cross-sectional study, Tan et al. (30) found that patients at risk of sarcopenia assessed by SARC-F had high levels of negative health outcomes, including recurrent hospital admissions, polypharmacy, multiple medical appointments, high rate of falls, and falls in clinical practice. Two previous studies have shown that Ishii's score can be used to predict future adverse events in patients with heart failure and chronic kidney disease (31,32) and was significantly associated with worse functional status overall (33). The current study results showed that SARC-F had good performance in predicting rehospitalization during a follow-up period of one year. The difference between this study and previous studies may be due to the following: first, the limited sample size in this study affected the power to detect differences between groups (sarcopenic *vs* nonsarcopenic) for the 1-year adverse outcomes using Ishii's score; second, the different patients selected; third, the five items of SARC-F focused on evaluating the physical function of patients and the Ishii's score, the calf circumference (on behalf of the lower limb muscle mass) and grip strength (on behalf of the upper limb muscle strength), which might be less representative of patients' functional statuses (34,35).

Herein, we also showed that SARC-F and CCI were independent risk factors for rehospitalization. However, rehospitalization was affected by several factors, and hence, evaluating the prognostic value of SARC-F should account for patient diseases. Gait speed served as a protective factor against rehospitalization. These results suggested that early exercise intervention for high-risk individuals detected by SARC-F might reduce the risk of adverse outcomes and improve prognosis.

The present study has several limitations. First, we used a convenience sample and the study probably lacked power to demonstrate associations with the other outcomes. Second, the follow-up time for adverse outcomes was only 1 year, and rehospitalization was the only outcome assessed by Cox analysis. Therefore, the conclusions cannot be extrapolated to the comprehensive and long-term prognostic value of the two screening tools, thereby necessitating a more prolonged follow-up period to establish prognostic value in the future. Third, this was an observational study for the existing tools, hence, developing new tools with better accuracy and prognostic ability is essential. Finally, the reference standard used in this study was based on the Consensus Report of AWGS that was conducted only in Chinese inpatients. Therefore, further studies are needed to evaluate both tools in populations other than Asians based on the same criteria.

In summary, both the SARC-F and Ishii's scores might facilitate the early detection of sarcopenia in clinical practice and, therefore, help clinicians to identify older adults at risk for adverse outcomes in acute settings.
